# The role of activity, scan duration and patient’s body mass index in the optimization of FDG imaging protocols on a TOF-PET/CT scanner

**DOI:** 10.1186/s40658-021-00380-9

**Published:** 2021-04-06

**Authors:** Roberta Matheoud, Naema Al-Maymani, Alessia Oldani, Gian Mauro Sacchetti, Marco Brambilla, Alessandro Carriero

**Affiliations:** 1 Medical Physics Department, Azienda Ospedaliero-Universitaria Maggiore della Carità, C.so Mazzini 18, 28100 Novara, Italy; 2 Department of Physics, University of Trieste, Via Valerio 2, 34127 Trieste, Italy; 3 Abdus Salam International Centre for Theoretical Physics (ICTP), Strada Costiera 11, 34151 Trieste, Italy; 4 Università del Piemonte Orientale, School of Medicine, V. Solaroli 17, 28100 Novara, Italy; 5 Nuclear Medicine Department, Azienda Ospedaliero-Universitaria Maggiore della Carità, C.so Mazzini 18, 28100 Novara, Italy; 6 Radiology Department, Azienda Ospedaliero-Universitaria Maggiore della Carità, C.so Mazzini 18, 28100 Novara, Italy

**Keywords:** Positron emission tomography, Time-of-flight, FDG, Contrast-to-noise ratio, Optimization, Image quality

## Abstract

**Background:**

Time-of-flight (TOF) PET technology determines a reduction in the noise and improves the reconstructed image quality in low count acquisitions, such as in overweight patients, allowing a reduction of administered activity and/or imaging time. However, international guidelines and recommendations on the ^18^F-fluoro-2-deoxyglucose (FDG) activity administration scheme are old or only partially account for TOF technology and advanced reconstruction modalities.

The aim of this study was to optimize FDG whole-body studies on a TOF-PET/CT scanner by using a multivariate approach to quantify how physical figures of merit related to image quality change with acquisition/reconstruction/patient-dependent parameters in a phantom experiment.

**Methods:**

The NEMA-IQ phantom was used to evaluate contrast recovery coefficient (CRC), background variability (BV) and contrast-to-noise ratio (CNR) as a function of changing emission scan duration (ESD), activity concentration (AC), target internal diameter (ID), target-background activity ratio (TBR) and body mass index (BMI). The phantom was filled with an average concentration of 5.3 kBq/ml of FDG solution and the spheres with TBR of 21.2, 8.8 and 5.0 in 3 different sessions. Images were acquired at varying background activity concentration from 5.1 to 1.3 kBq/ml, and images were reconstructed for ESD of 30–151 s per bed position with and without point spread function (PSF) correction. The parameters were all considered in a single analysis using multiple linear regression methods.

**Results:**

As expected, CRC depended only on sphere ID and on PSF application, while BV depended on sphere ID, ESD, AC and BMI of the phantom, in order of decreasing relevance. Noteworthy, ESD and AC resulted as the most significant predictors of CNR variability with a similar relevance, followed by the BMI of the patient and TBR of the lesion.

**Conclusions:**

AC and ESD proved to be effective tools in modulating CNR. ESD could be increased rather than AC to improve image quality in overweight/obese patients to fulfil ALARA principles.

## Background

Thanks to the improvements in hardware components and in imaging reconstruction techniques, significant advances have been made in recent years in positron emission tomography/computed tomography (PET/CT) systems [1, 2]. They are mainly related to the use of fast detectors, lutetium oxyorthosilicate (LSO) and/or lutetium-yttrium oxyorthosilicate (LYSO) coupled to both time-of-flight (TOF) technology and advanced reconstruction modalities such as the modelling of the system point spread function (PSF) [3–5] and/or noise [6] which improve the accuracy of quantitative information and enhance the detectability of small lesions [7, 8].

The main motivation for TOF-PET has always been the potential image quality improvement or reduction in image acquisition time [1, 2]. The effective sensitivity gain was already described nearly 40 years ago [8, 9] as depending on the ratio between the object size *D* and the spatial FWHM of the TOF kernel Δ*x*. This improvement has been used in the clinical setting predominantly to reduce the imaging time [1, 2].

PET imaging of larger patients is affected by high noise levels because of the considerable intrinsic attenuation and in clinical practice typically a longer acquisition time is needed to compensate for the poor quality of the data. TOF technology acts as a compensation, bringing the image quality in larger patients closer to that in patients of average size [10, 11]. Thus, the first consequence of TOF technology is the increase in SNR gain, this being more evident for larger patients [2, 12].

However, international guidelines and recommendations on ^18^F-fluoro-2-deoxyglucose (FDG) activity administration scheme are rather old or only partially account for time-of-flight technology [13] and advanced reconstruction modalities. Further indications on optimization were provided by EARL [14] that suggested a procedure to assess to which level of FDG activity can be reduced while keeping image quality and quantification accuracy within acceptable limits. Other studies recommended a quadratic relation between the FDG activity to administer and the body mass of the patient [15] or criteria based on the NECR-dosage curve [16].

However, precise information on how to tune administered FDG activity and emission scan duration in whole-body oncological studies on TOF-PET/CT scanners is still a demanding need for the nuclear medicine physicians.

Few papers in the literature studied the optimization of FDG activity administration [17, 18] or emission scan duration [19] on TOF-PET/CT scanners, but they examined the two factors independently.

The aim of this work was to describe how the physical figures of merit related to PET image quality change with different acquisition, reconstruction and object-dependent parameters on a TOF-PET/CT scanner. The study was designed to simultaneously analyse the impact of the different factors with a multivariable approach, using phantoms with a variable weight, which hosted several well-defined target sizes with a known target-to-background ratio, as done in a previous study [20]. We selected the emission scan duration (ESD), the FDG activity concentration (AC), the target-to-background activity concentration ratio (TBR), the target size (ID), the body mass index (BMI) of the scanned object and the application of PSF correction (PSF) as the factors that could affect the contrast recovery coefficient (CRC), the background variability (BV) and the contrast-to-noise ratio (CNR), identified as PET image quality descriptors.

## Methods

### PET/CT scanner

The Ingenuity TF 64 (Philips Healthcare, Cleveland, OH, USA) is a lutetium-yttrium oxyorthosilicate (LYSO) TOF-PET/CT scanner. The energy window is set between 440 and 665 keV, the coincidence window is 3.8 ns and the TOF resolution is 536.2 ps.

The performance characteristics of this TOF-PET/CT scanner according to NEMA NU 2012 standard have already been described in detail [21].

### Phantom setup

**Fig. 1:**
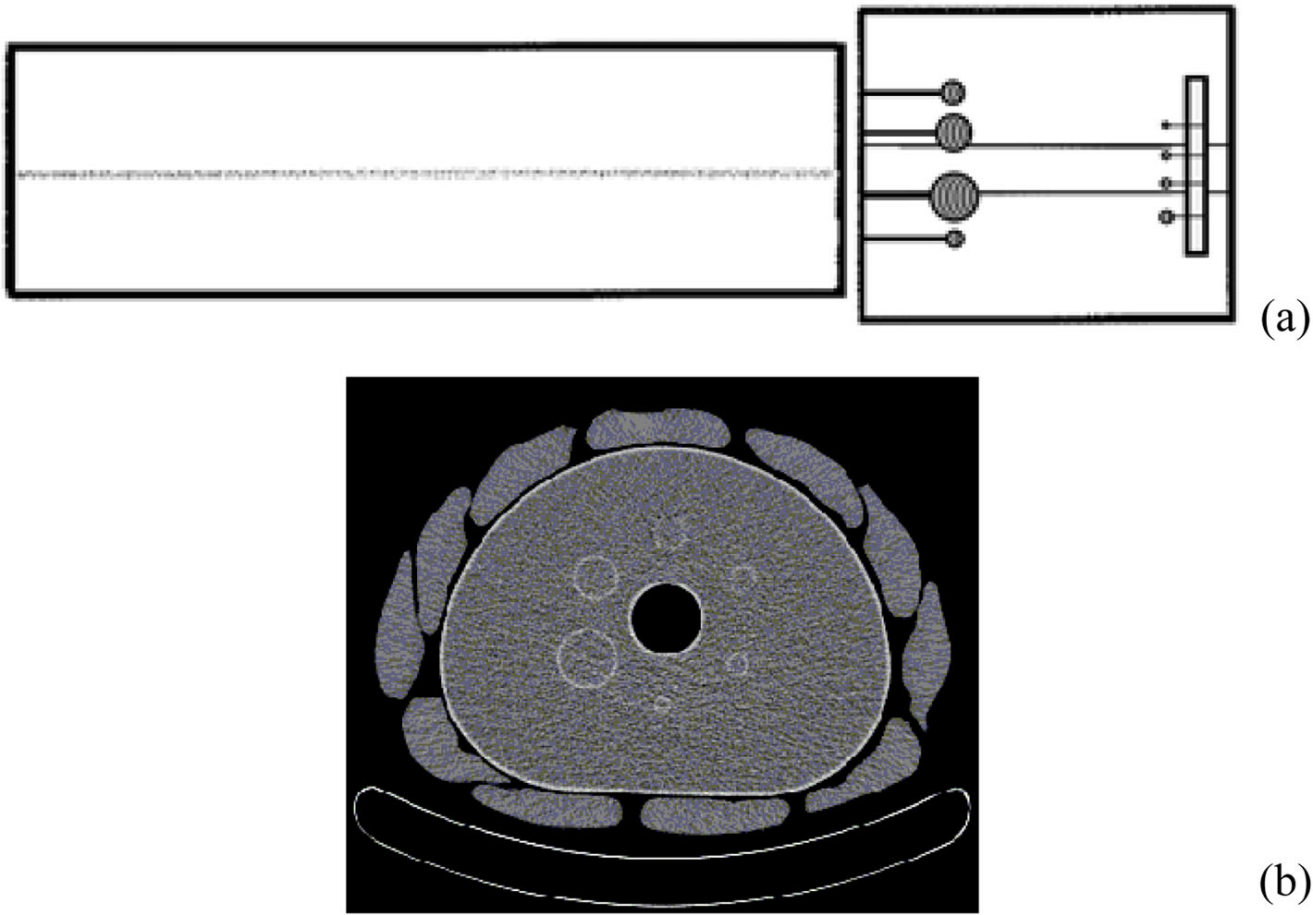
Phantom setup in antero-posterior view (**a**) and CT scan of the b-NEMA-IQ phantom at standard spheres level (**b**)

The International Electrotechnical Commission (IEC) 61675–1 emission phantom (NEMA image quality phantom, NEMA-IQ phantom) with FDG solution was used. The NEMA-IQ phantom has an interior cavity volume of 9947 ml and contains 6 fillable spheres with 10, 13, 17, 22, 28 and 37 mm inner diameters (ID). A cylindrical insert filled with low-density foam (density of 0.30 g/cm^3^) was fixed along the centre of the phantom. Four micro-hollow fillable spheres with ID of 4.1, 4.7, 6.5 and 8.1 mm (Data Spectrum Corporation) were inserted in the NEMA-IQ phantom to simulate smaller lesions. The microspheres were tightly fixed to a foam support attached to the lung insert at the bottom of the phantom (Fig. 1a) in a unique position that guarantees the microspheres to be set always at the same height with respect to the base of the phantom to exclude possible bias in the further analysis.

The NEMA-IQ phantom was centred in the transverse FOV of the scanner with the equatorial plane of the standard spheres coplanar to the centre of the axial FOV.

To simulate the activity outside the scanner FOV, the scatter phantom (Data Spectrum Corporation) was placed close at the end of the IEC phantom, strictly following NEMA NU-2 recommendations [22]. It is a solid circular cylinder composed of polyethylene with an outside diameter of 203 mm and a length of 700 mm. A 6.4-mm hole is drilled along the central axis of the cylinder. A 700-mm polyethylene tube with an inside diameter of 3.2 mm and an outside diameter of 4.8 mm is placed in the hole.

Finally, to simulate a different patient habitus, a ‘belt’ of 11 water bags of 500 ml and 3 cm thick was fit over the NEMA-IQ phantom (Fig. 1b) [20, 23]. A few papers in the literature [24, 25] developed methods to assess the size-specific dose estimate in patients undergoing chest and abdomen CT examinations, based on the relation between the body mass index (BMI) and the effective diameter *d*_eff_ of the patient:

1 $$ {\mathrm{d}}_{\mathrm{eff}}\left(\mathrm{cm}\right)=0.6414\ \left(\mathrm{cm}\cdotp {\mathrm{m}}^2/\mathrm{kg}\right)\mathrm{x}\kern0.5em \mathrm{BMI}+12.976\ \left(\mathrm{cm}\right) $$

BMI was calculated accordingly to the formula provided by the World Health Organization [26], BMI = (weight × height^−2^) and *d*_eff_ = $\sqrt{d_{LL}{d}_{AP}}$, being *d*_LL_ and *d*_AP_ the latero-lateral and antero-posterior diameter of the patient.

From the measure of the *d*_AP_ and *d*_LL_ made on the CT scan of both NEMA-IQ and NEMA-IQ fitted with the additional belt, BMI values were calculated by means of the relation above for the two configurations.

### Phantom preparation and acquisition

The spheres of the NEMA-IQ phantom were filled with ^18^F activity concentrations of 117.3, 46.6 and 25.6 kBq/ml and the main compartment with ^18^F activity concentration of 5.5, 5.3 and 5.1 kBq/ml, in three different experimental sessions, respectively, thus providing 21.2, 8.8 and 5.0 target-to-background ratios (TBRs). The reference time for all the activity concentrations is the time of the first acquisition.

A more clinical parameter used to evaluate the uptake in a lesion is the standardize uptake value (SUV) which describes the activity concentration in the lesion with respect to the total activity concentration in the phantom (this last being normally higher than the background concentration). The correspondent theoretical SUV values realized in the three experimental sessions were 19, 8 and 5, respectively.

The capillary present in the scatter phantom was filled with ^18^F activity concentrations of 5.3, 5.1 and 5.3 kBq/ml in the three experimental sessions, respectively.

PET/CT raw data of both NEMA-IQ and NEMA-IQ wrapped with the belt (b-NEMA-IQ) were acquired in list mode following NEMA NU-2 2012 protocol [22]. NEMA-IQ and b-NEMA-IQ phantoms were acquired sequentially, at different activity concentrations in the main compartment of about 5.1, 3.1, 2.2, 1.5 and 1.3 kBq/ml. Overall, 30 (5 activity concentrations × two phantoms × 3 sessions) acquisitions were performed. A CT scan was used for attenuation correction.

### Image reconstruction

Images were reconstructed using a TOF, list-mode, blob-based, ordered subsets maximum likelihood expectation maximization algorithm (TOF-OSEM) [27] by applying correction for attenuation, scatter, random, detector normalization, radioactive decay, system dead time and crystal timing.

The standard protocol provided by the manufacturer for clinical whole-body examinations was used to reconstruct all the images acquired, by setting the Speed to Normal (99 equivalent iterations and a TOF kernel width of 14.1 cm) and the Smooth to Normal (full width at half maximum (FWHM) of the Gaussian filter equal to 4 mm and the relaxation parameter equal to 1.0) on a 144 × 144 frame (4 mm isotropic voxel). The reconstructions were performed for different emission scan durations (ESD) with (PSF = 1) and without (PSF = 0) the application of the resolution recovery algorithm or PSF correction (PSF). On the Ingenuity TF PET/CT scanner, PSF correction is characterized by two parameters, the number of iterations and the regularization kernel width (FWHM expressed in mm). The default parameters suggested by the manufacturer (iteration = 1, regularization = 6 mm) were used when PSF was applied.

The different ESD were obtained by cutting the list-mode file after ESD seconds. Namely, ESD was set to 30, 45, 60, 75, 90 and 120 s. The PSF, speed and smoothing filter parameters were kept fixed for each reconstruction, as no significant difference in contrast recovery coefficient and background variability exists by changing speed, smooth and PSF values [21].

### Image analysis

The percentage contrast recovery coefficient (CRC) and the background variability (BV) were evaluated by a routine provided by the manufacturer, according to NEMA NU-2 2012 standards:

2 $$ {\mathrm{CRC}}_j=\frac{\left(\frac{C_{\mathrm{S}j}}{C_{\mathrm{B}j}}\right)-1}{\left(\frac{A_{\mathrm{S}}}{A_{\mathrm{B}}}\right)-1}\times 100\% $$

3 $$ {\mathrm{B}\mathrm{V}}_j=\frac{SD_j}{C_{\mathrm{B}j}}\times 100\% $$

where $$ {SD}_j=\sqrt{\sum_1^k{\left({C}_{\mathrm{B} jk}-{C}_{\mathrm{B}j}\right)}^2/\left(k-1\right)} $$

*C*_S*j*_ = average counts/s in the ROI for sphere *j*

*C*_B*j*_ = average of the background counts/s for sphere *j*

*A*_S_ = activity concentration in the spheres

*A*_B_ = activity concentration in the background

This routine provides the user with a pattern of six ROIs of fixed diameters equal to the physical internal diameter of the spheres and fixed relative distances. After choosing the slice corresponding to the equatorial section of the spheres, the user can only rotate and translate the pattern to establish its correct position over the hot spheres in the central slice. A pattern of twelve groups of 37-mm background ROIs at a distance of 15 mm from the edge of the phantom but no closer than 15 mm to any sphere is linked to the pattern of the six ROIs and is automatically placed in the background. The positioning of the smaller ROIs (10, 13, 17, 22 and 28 mm) on background, concentric to the 37 mm ones, is done automatically. The same pattern of 12 background ROIs is automatically positioned at a distance of ±1 and ± 2 cm from the central slice for a total of *K* = 60 background ROIs, as prescribed by NEMA recommendations. The analysis of CRC and BV for the four microspheres was performed by means of a routine developed on purpose in ImageJ v 1.48 (National Institutes of Health, Bethesda, MD, USA [28]). The ImageJ routine was written to act similarly to the manufacturer’s one, with the difference that the pattern provided contained 4 ROIs of fixed diameters equal to the physical internal diameter of the microspheres and fixed relative distances correspondent to their positions. The background ROI pattern consisted of 12 groups of concentric ROIs of 4.1, 4.7, 6.5 and 8.1 mm diameters.

Both routines output CRC and BV values for each sphere and ROI dimension, as well as the mean and standard deviation of the activity concentration for all the ROIs drawn.

Moreover, the contrast-to-noise ratio (CNR), which is the physical figure of merit more closely related to lesion detectability, was evaluated for all the spheres that were detected on the reconstructed images. In accordance with [20], CNR for sphere *j* was defined as:

4 $$ \mathrm{CNR}j=\frac{\left({C}_{\mathrm{S}j}-{C}_{\mathrm{B}}\right)/{C}_{\mathrm{S}j}}{SD_{\mathrm{B}}/{C}_{\mathrm{B}}} $$

where *C*_S*j*_ = average counts in the ROI for sphere *j*

${C}_{\mathrm{B}}=\frac{1}{J}{\sum}_1^J{C}_{\mathrm{B}j}$, *C*_B*j*_ is the average counts measured in all background ROIs of size *j*,

${SD}_{\mathrm{B}}=\sqrt{\sum_1^J{\sum}_1^K{\left({C}_{\mathrm{B}j,k}-{C}_{\mathrm{B}}\right)}^2/\left(K\bullet J-1\right)}$, *J* = 8 is the standard deviation of *C*_B*j*,*k*_ (accounting for the number of visible spheres) and *K* = 12 (accounting for the ROI positions in the phantom background).

Overall, 3360 CNR values were evaluated (8 visible ID × 7 ESD × 3 TBR × 5 AC × 2 phantoms × 2 PSF values).

Finally, the visibility of the hot spheres in the reconstructed images was assessed independently by two nuclear medicine physicians using a 5-point rating scale from 0 to 4 to answer the question “Is the sphere visible?” The scores were defined as 0 = definitely no, 1 = probably no, 2 = possibly yes, 3 = probably yes and 4 = definitely yes. Three sets of consecutive transaxial image planes were displayed for each case to be rated, allowing the reader to evaluate the central plane in the context of adjacent planes. Readers were able to adjust the lookup table of each image to facilitate image viewing. The order in which image sets were evaluated was randomized.

### Statistical analysis

Correlation matrices were used to identify potential univariate correlations between image quality figures of merit (CRC, BV and CNR) and acquisition-dependent (ESD, AC), reconstruction-dependent (PSF application) and object-dependent parameters (ID, TBR and BMI). Only significant predictors at univariate analysis were considered and inserted into multiple linear regression methods to derive analytical formulas of the model.

The weight of different independent variables in explaining the dependent variables was quantified by means of standardized regression coefficients (*β*). The *β*s can be used as a measure of relative importance, with the independent variables ranked in order of the sizes of these coefficients (ignoring sign) [29].

Box and whisker plots were used to provide a univariate graphical representation of CRC and BV with respect to significant predictors, identified by the regression models.

The impact of the different acquisition- and object-dependent parameters on CNR was further investigated by a multiple-way principal effects ANOVA: acquisition- and object-dependent parameters were considered as independent variables (factors) and CNR as the dependent variables A post hoc test (Scheffè *F* test) was performed to identify the main sources of variability. If a significant *F* value was found for one independent variable, then this was referred as a main effect. When a main effect was found, then the Scheffè test was performed to compare the dependent variable upon the levels of the factor 2 × 2, thus identifying the main sources of variability. These comparisons were represented by drawing the least squares means, which are the best linear estimates for the marginal means in the ANOVA design, together with the standard errors of the means (and thus the 95% confidence intervals) [29]. The reproducibility of visual scoring of hot spheres in the reconstructed images was estimated by the correlation agreement using Cohen weighted kappa (*k*_W_).

All statistical analyses were performed with the software STATISTICA 6.0 (Statsoft Inc, USA).

## Results

### Phantoms

The *d*_AP_ and *d*_LL_ measured on the CT scan were 22.9 and 29.5 cm and 27.9 and 36.1 cm for the NEMA-IQ and the b-NEMA-IQ phantoms, respectively, resulting in effective diameters of 26.0 and 31.7 cm, respectively (Fig. 1b). By using relation (1), the *d*_eff_ values corresponded to BMI values of 21.4 and 29.2 kg/m^2^ for NEMA-IQ and b-NEMA-IQ, respectively, and allow to classify the two phantoms as simulating normal and high-overweight patients.

### Contrast recovery coefficient

The recovery of ^18^F activity in the spheres of NEMA-IQ phantom depends on sphere ID (*β*_ID_ = 0.68) and on the application of PSF correction (*β*_PSF_ = 0.23), in order of decreasing relevance of the weight of the variable in the model. *F* was 1382 (*p* < 0.0001). The adjusted *R*^2^ of model fitting was 0.51 and the residuals were normally distributed.

**Fig. 2:**
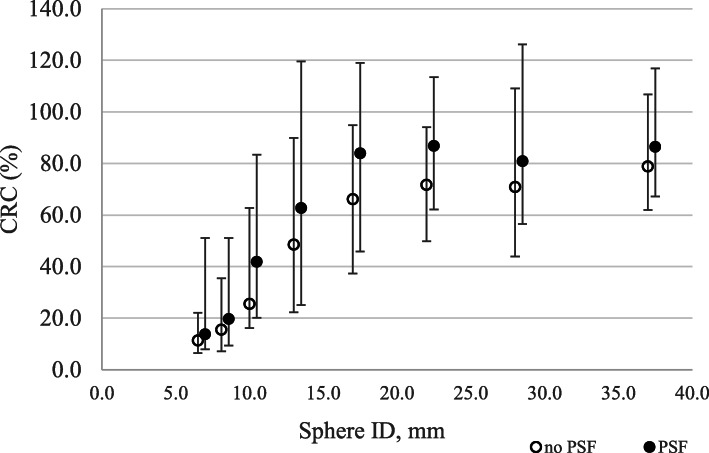
Box plots of CRC values as a function of sphere ID and PSF for visible spheres. Outliers and extremes are points higher than the value of the 75th percentile plus 1.5 or 3 times the interquartile distance, or lower than the value of the 25th percentile minus 1.5 or 3 times the interquartile distance, respectively

Figure 2 shows a box plot of CRC with respect to sphere ID and PSF correction for visible spheres.

### Background variability

The multiple linear regression analysis showed that BV depends on sphere ID (*β*_ID_ = − 0.65), ESD (*β*_ESD_ = − 0.40), AC (*β*_AC_ = − 0.35) and *W* (*β*_W_ = 0.25), in order of decreasing relevance. *F* was 1641 (*p* < 0.0001). The adjusted *R*^2^ of model fitting equals 0.71 and the residuals were normally distributed. The multiple linear regression equation that summarizes the model with BV as predicted variable and sphere ID, ESD, AC and BMI as predictors can be written as:

5 $$ \mathrm{BV}=23.7-\left(0.5{\mathrm{m}\mathrm{m}}^{-1}\times \mathrm{sphere}\ \mathrm{ID}\right)-\left(0.1{\mathrm{s}}^{-1}\times \mathrm{ESD}\right)-\left(1.8\frac{\mathrm{m}\mathrm{l}}{\mathrm{kBq}}\times \mathrm{AC}\right)+\left(0.4\frac{{\mathrm{m}}^2}{\mathrm{kg}}\times \mathrm{BMI}\right) $$

**Fig. 3:**
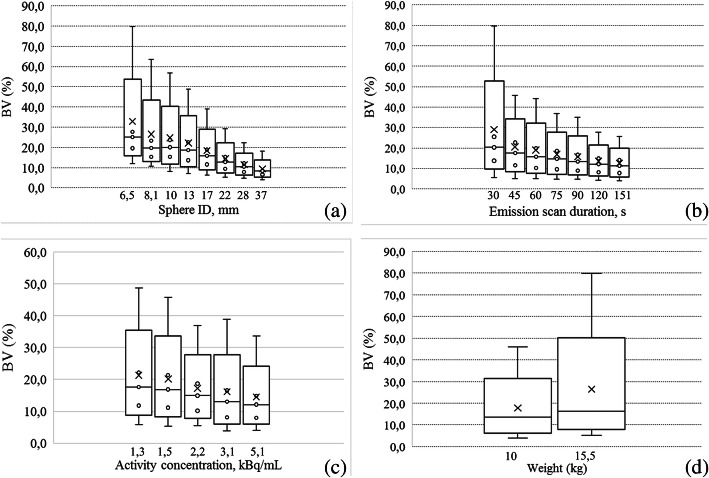
Box plots of BV as a function of sphere ID (**a**), ESD (**b**), AC (**c**) and W (**d**) for visible spheres. Outliers and extremes are points higher than the value of the 75th percentile plus 1.5 or 3 times the interquartile distance, or lower than the value of the 25th percentile minus 1.5 or 3 times the interquartile distance, respectively

Figure 3 shows a box plot of BV with respect to sphere ID (a), ESD (b), AC (c) and BMI (d), respectively, for visible spheres.

### Contrast-to-noise ratio

The CNR of the spheres detected on the images depends on ESD (*β*_ESD_ = 0.53), AC (*β*_AC_ = 0.50), BMI (*β*_W_ = −0.37) and TBR (*β*_ESD_ = 0.26), in order of decreasing relevance. *F* was 1528 (*p* < 0.0001). The adjusted *R*^2^ of model fitting equals 0.73 and the residuals were normally distributed. The regression equation that best summarizes the results obtained in a multiple regression model for CNR is:

6 $$ \mathrm{CNR}=4.87+\left(0.03{\mathrm{s}}^{-1}\times \mathrm{ESD}\right)+\left(0.74\frac{\mathrm{m}\mathrm{l}}{\mathrm{kBq}}\times \mathrm{AC}\right)-\left(0.19\frac{{\mathrm{m}}^2}{\mathrm{kg}}\times \mathrm{BMI}\right)+\left(0.08\times \mathrm{TBR}\right) $$

**Fig. 4:**
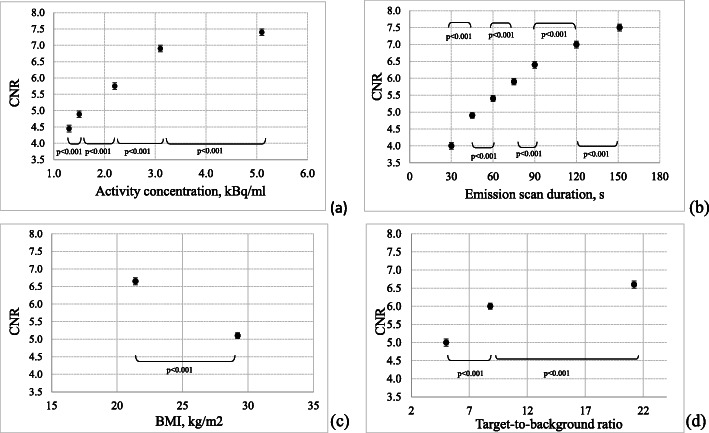
CNR as a function of AC (**a**), ESD (**b**), W (**c**) and TBR (**d**). Points represent least square averages; vertical bars represent 95% confidence intervals. The results of the Scheffè test are displayed for the adjacent levels of the predictor variables

ESD and AC impact with a similar weight on CNR. As expected, BMI impacts with a negative regression coefficient on CNR, i.e. as the BMI increases the CNR decreases. Only last came TBR, with a slight impact on CNR about one half the one of ESD and AC. The major gain in CNR was observed for low TBR (or low SUV) values, as when moving from TBR 5.0 to 8.8 the CNR increases of about 19%, while when moving from TBR 8.8 to 21.2, the CNR increase is only of about 9% (Fig. 4d). However, this is an intrinsic characteristic of the lesion itself and cannot be managed in the optimization process.

Post hoc Scheffè test showed a statistically significant increase in CNR for every contrast between adjacent levels of AC in the range explored (*p* < 0.001) (Fig. 4a). When considering 2.2 kBq/ml, which represents the activity concentration 60 min post-injection of 3 MBq/kg of FDG, the CNR mean value increases of about 19% when moving to an activity concentration of 3.1 kBq/ml, which corresponds to an injection scheme of 4.5 MBq/kg (in the hypothesis of a density of 1 kg/dm^3^ or equivalently 1 g/cm^3^).

A similar behaviour was observed for all the ESD, BMI and TBR contrast tested. Post hoc Scheffè test showed a statistically significant increase in CNR for every contrast between adjacent levels of ESD (Fig. 4b), BMI (Fig. 4c) and TBR (Fig. 4d) in the range explored (*p* < 0.001).

### Visual detection of the spheres

**Fig. 5:**
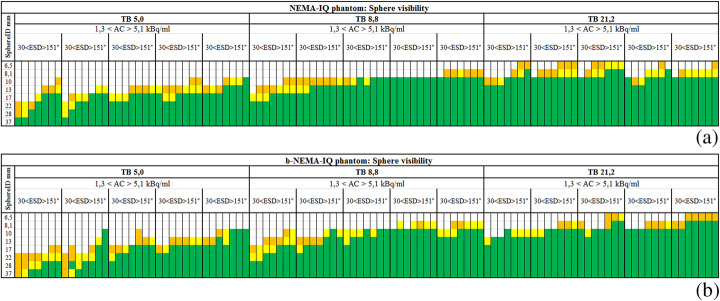
Result of the visual detection of the spheres for NEMA-IQ (**a**) and b-NEMA-IQ (**b**) phantoms for different TBR, AC and ESD and sphere ID, in terms of median scores of the two observers: white [0–2.0], orange [2.0–2.7], yellow [2.7–3.4] and green [3.4–4.0]

Interobserver reproducibility analysis showed an excellent reproducibility between the two observers (*k*_W_ = 0.87). A hot sphere was considered visually detectable if its median score was 2. Figure 5 a and b show the results of visual detection of different sphere ID for different TBR, AC and ESD for NEMA-IQ and b-NEMA-IQ phantoms, respectively. From these data, it is evident that the overall scores for the lesions in the NEMA-IQ phantom are higher than those for the lesions in the b-NEMA-IQ one, confirming the impact of the BMI on lesion detectability. The difference in the scores of visual detection is particularly evident for low TBR and low activity concentrations and for intermediate TBR and low activity concentrations.

In particular, one can observe that this PET/CT scanner is unlikely to detect lesions with a dimension of 6.5 mm or less with TBR 21.2 or lower, lesions of dimension 8.1 mm or less with TBR is 8.8 or lower, and lesions of a dimension of 13 mm or less with TBR ratio is 5.0 or lower. Moreover, the increase in activity concentration and emission scan duration appears to be particularly important to achieve lesion detection for the b-NEMA-IQ phantom configuration.

**Fig. 6:**
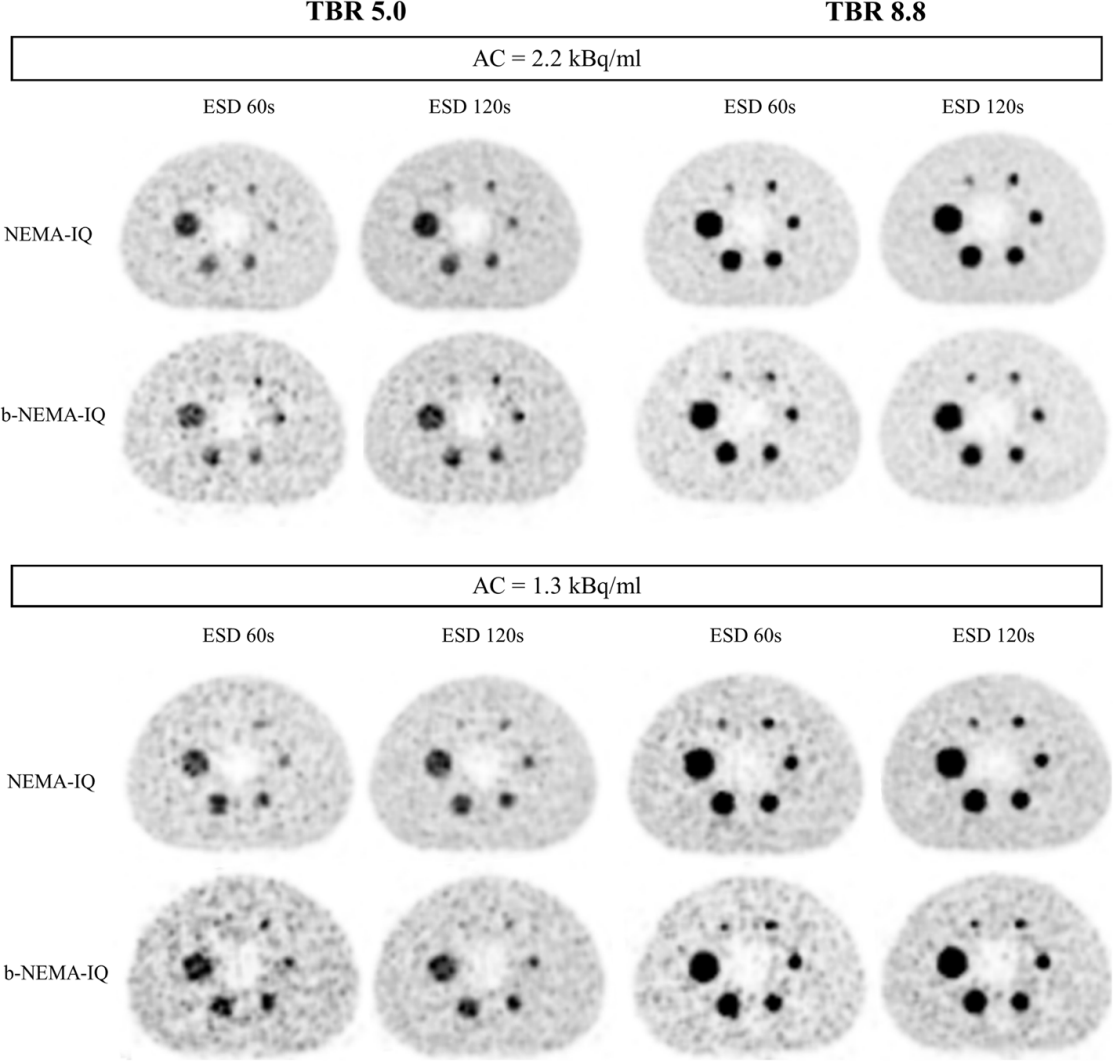
Equatorial slices of standard spheres of NEMA-IQ and b-NEMA-IQ phantoms for low (5.0) and intermediate (8.8) TBR, two values of AC (1.3 and 2.2 kBq/ml) and two values of ESD (60 and 120 s). The impact of increasing ESD or AC is evident on both the reduction of noise in the background and in the greater visibility of the 10-mm sphere

Figure 6 shows reconstructed images of standard spheres of NEMA-IQ and b-NEMA-IQ phantoms for low and intermediate TBR values, two AC and ESD values.

**Fig. 7:**
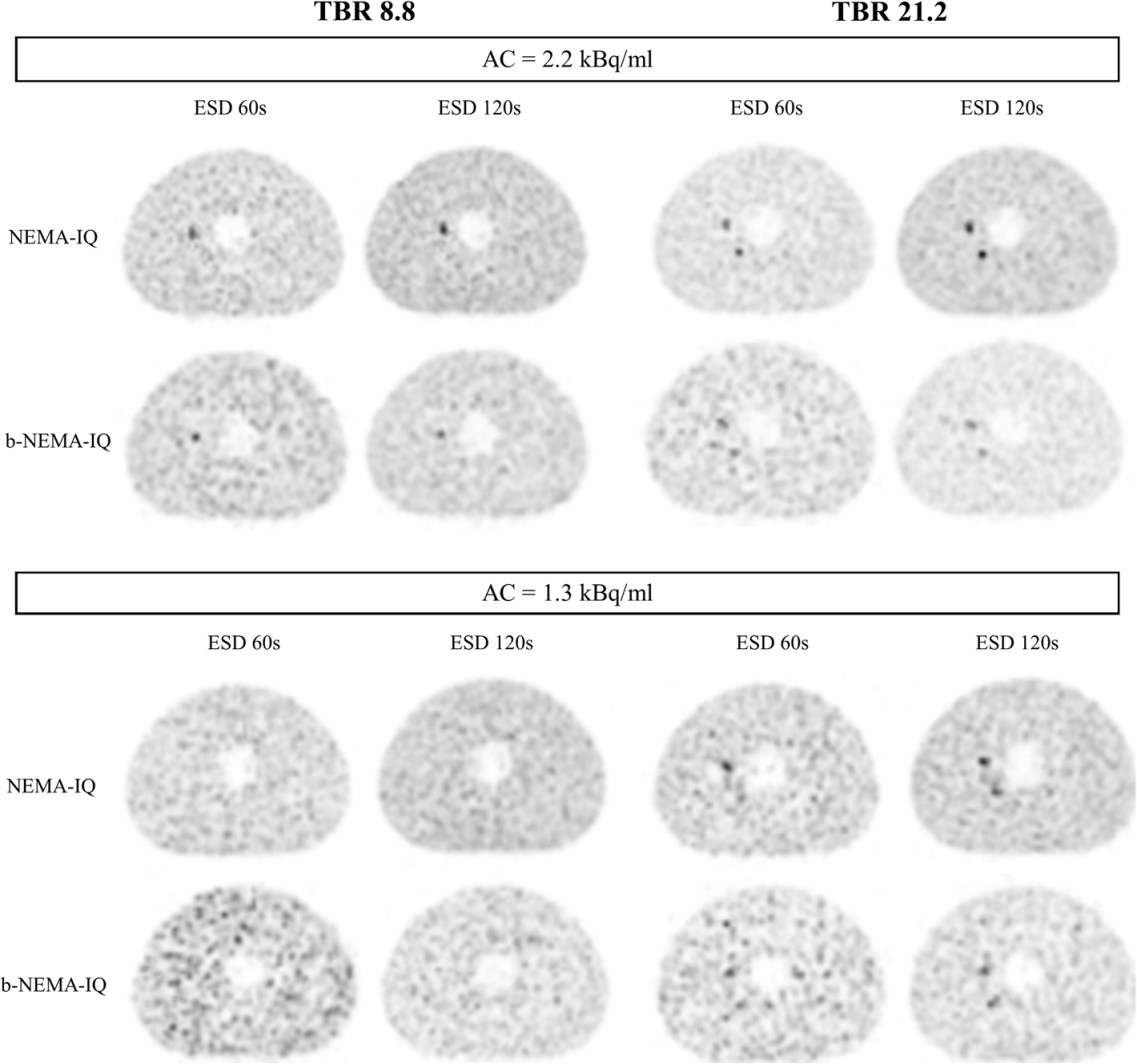
Equatorial slices of microspheres for NEMA-IQ and b-NEMA-IQ phantoms for intermediate (8.8) and high (21.2) TBR, two values of AC (1.3 and 2.2 kBq/ml) and two values of ESD (60 and 120 s). The impact of increasing ESD or AC is evident in the greater visibility of the 8.1-mm microsphere. The increase of TBR allows visibility of the 6.5-mm microsphere. At intermediate TBR (8.8) and low AC (1.3 kBq/ml), no microsphere is detectable

Figure 7 shows reconstructed images of microspheres of NEMA-IQ and b-NEMA-IQ phantoms for intermediate and high TBR values, two AC and ESD values.

## Discussion

The optimization process invoked by the Euratom Directive 2016-59 [30] requires optimal image quality of FDG whole-body PET/CT examinations by opportunely tuning acquisition and reconstruction parameters as well as the FDG activity to administer to patients. This process must be performed in the light of reducing the radiation dose burden in particular to patients frequently exposed to several radiological examinations during their follow-up. The image quality and lesion detectability in FDG PET imaging are limited by different factors as the low signal-to-noise ratio, the relatively low spatial resolution and patient’s motion, which results in the partial-volume effect affecting lesion visualization and quantitation [2].

This work was aimed to characterize the quality of PET images for a TOF-PET/CT scanner in a wide range of acquisition, reconstruction and object-dependent parameters in settings like those encountered in clinical practice, by means of a phantom study. CRC, BV and CNR, which are closely related to lesion detectability, were the figures of merit used to describe PET image quality. Our study used a multivariate approach to quantify how these figures of merit change as a function of ESD and AC for different target sizes, TBR, BMI and under the effect of the point spread function correction.

The main result of this study is that the CNR of FDG lesions depends on ESD and AC in a similar way (*β* = 0.53 and *β* = 0.50). A previous study performed on a non-TOF-PET scanner [20] concluded that the main predictor of CNR was ESD (*β* = 0.60) and only with a half of the explanatory power (*β* = 0.27) came AC. On the other hand, EANM guidelines [13] provide administration schemes based on a linear and a quadratic relationship [15], respectively, between PET acquisition time per bed position, patient weight and recommended FDG activity. Moreover, the difference in CNR dependence on ESD and AC on the two PET/CT scanners may rely on the TOF technology that is available in the PET/CT scanner used in the present study, but not in [20]. As the reduction of acquisition time is one of the main achievements of TOF technology, the present study was performed by using smaller emission scan durations and in a narrower range with respect to those used in [20]. This last can be the reason for the reduced dependence of CNR on ESD with respect to non-TOF-PET scanners. Moreover, our results show also a significant increase in CNR for each increasing step in AC or ESD in the range explored, i.e. from 1.3 to 5.1 kBq/ml and from 30 to 151 s. The visual inspection of phantom images confirmed that the image quality, in terms of the noise level and contrast, can be improved by increasing the AC (Fig. 4a) or ESD (Fig. 4b). This finding agrees with the clinical results reported in the recent paper of Prieto [18]. The author observed a statistically significant difference in both the image noise and the overall image quality indexes of PET images obtained after FDG activity administration of 5.2 and 3.7 MBq/kg on the Siemens mCT TOF-PET/CT scanner.

The third predictor of CNR was the BMI of the phantom (*β* = −0.37), indicating that on average the CNR decreased by 25% with increasing the BMI of the scanned object by a factor of 1.4. It is known that the TOF technology allowing the reduction of the uncertainty on the annihilation event acts as a noise equalizer and brings an overall gain in signal-to-noise ratio, being this effect more evident for larger objects [1, 10]. However, notwithstanding TOF technology, this result confirms that there is still a residual dependence of CNR on the size of the imaged object as evidenced by the literature [1]. The b-NEMA-IQ phantom realized in this study, with a BMI of 29.6 kg/m^2^ calculated from its *d*_eff_, simulates a high-overweight patient. A way to improve lesion detection is then to define specific acquisition protocols for oncological whole-body studies tailored on patient’s BMI, rather than using a fixed ESD. Should this result be confirmed in clinical studies, it would indicate an additional way to improve the lesion detectability for larger patients, depending on their weight or their body habitus.

The last predictor of CNR was the TBR of the lesion (*β* = 0.26). This dependence may be explained by considering that the complete recovery of the true amount of the activity concentration in a lesion is hampered at low values of TBR. By increasing the TBR value, the recovery of the activity concentration improves, and the contrast-to-noise ratio increases accordingly.

The fitted multiple regression model of CNR based on these premises accounts for more than two thirds of CNR variance (adjusted *R*^2^ = 0.76).

From these findings, one can derive that it is possible to opportunely tune ESD and AC on patient’s BMI in order to keep constant the CNR, or the detectability level, on the PET images of this scanner.

As a typical example, let us consider the following situation: a lesion with a TBR of 9 (SUV = 8.8) in a standard BMI patient injected with 3 MBq/kg of FDG imaged for 60 s, 60 min post-injection. According to Eq. (4), we should expect a CNR of 5.9 for a 10-mm-diameter lesion. To obtain a similar value in CNR for the same lesion uptake in a high-overweight patient injected with 3 MBq/kg of FDG, the patient should be scanned for 110 s. In an analogous way, one could double the activity administration scheme (i.e. 6 MBq/kg) to obtain the same CNR. However, from a radiation protection point of view, it would be more advisable to increase the ESD than the AC for an improvement in lesion detectability, still considering that a long ESD would increase the risk of patient’s movement.

Another result of this study is the dependence of CRC on sphere ID (*β* = 0.68) and PSF application (*β* = 0.23). This result confirms the dependence of CRC already reported by Zorz et al. [21], even with a slightly different analytical expression with respect to (2). This may be explained by observing that in [21] only four spheres (ID = 10, 13, 17 and 22 mm) were included in the NEMA-IQ phantom analysis and CRC fitting was almost perfectly linear (*R*_adj_^2^ = 0.93). In our study, on the contrary, CRC dependence on ID was analysed in a wider sphere dimension range, where CRC values assume a trend with respect to ID (Fig. 2) definitely different from the linear one, explaining also the relatively low *R*_adj_^2^ of 0.51.

The third parameter related to image quality we investigated, the BV resulted to be dependent on the ROI dimension for which is defined (*β* = −0.62), emission scan duration (*β* = −0.39), activity concentration (*β* = −0.31) and BMI (*β* = 0.24), in order of decreasing relevance, this model explaining 77% of variance in BV (*R*_adj_^2^ of 0.77). The strong dependence of BV on sphere ID is not new and was also found by Zorz et al. [21] and Brambilla et al. [20], with similar weights. Even if BV cannot be considered a descriptor of noise, it is interesting to note the equivalent dependence of BV on ESD (*β* = −0.39) and AC (*β* = −0.31), in analogy with the finding that CNR depends on both these parameters with similar weights, reported above. The impact of the BMI of the phantom on BV reinforces the same finding on CNR.

Some limitations of the present study must be acknowledged.

First, the results on CNR, CRC and BV found in the present study strictly apply to this PET/CT scanner and to 18F whole-body acquisitions: extrapolation of these results to different TOF-PET/CT scanners and to different radionuclides should be tested in advance before application. This consideration is even strengthened when considering digital state-of-the-art TOF-PET scanners for which the improvement in time resolution, namely in the range 214–380 ps [31], results in a reduction of noise. The consequent decrease in both AC and ESD reported in the literature [32, 33] would thus influence the relations between these parameters and the image quality figures of merit found in the present work.

Second, the ‘belt’ added to the NEMA-IQ phantom to simulate a high-overweight patient was filled with non-radioactive water, while in obese patients, the adipose tissue is mildly radioactive, typical SUVs being around 0.3 g/ml. When considering the attenuation and the scatter caused by the belt on the photons coming from the NEMA-IQ phantom, these effects would be equivalent to those originating from a 18F filled belt. Nevertheless, the photons emitted from the radioactive belt will cause an increase in the noise and thus a deterioration of the quality of the NEMA-IQ phantom image.

Another issue concerning the ‘belt’ is the presence of air gaps between the water bags and the NEMA-IEC phantom, as Fig. 1 shows, the air gaps accounting for less than the 10% of the total area of the section of the b-NEMA-IEC phantom, reducing the attenuation and the scatter phenomena effects. In the ultimate analysis, both the non-radioactive water and the presence of air gap between the ‘belt’ and the NEMA-IQ phantom are in some way an ameliorative condition of the real clinical situation. Thus, in this scenario, the conclusion that the image quality in overweight patients is worsened is reinforced.

Third, the NEMA-IQ and the b-NEMA-IQ phantoms represent the thorax-abdominal regions of a patient. For a comprehensive analysis of the effect of activity concentration, emission scan duration and target-to-background ratio on image quality, the simulation of the head and neck and pelvis regions should be devised. To this end, clinical studies would help in confirming the results of the present study.

## Conclusion

Activity concentration and emission scan duration proved to be effective tools in modulating the lesion detectability in FDG whole-body examinations.

Moreover, notwithstanding TOF technology, this study confirms that there is still a residual dependence of the contrast-to-noise ratio on the size of the imaged object, thus indicating that a way to improve lesion detection is to define specific acquisition protocols for oncological whole-body studies tailored on patient’s BMI, rather than using a fixed ESD.

## Data Availability

The datasets used and analysed during the current study are available from the corresponding author on reasonable request.

## Abbreviations

TOF: Time-of-flight
FDG: ^18^F-fluoro-2-deoxyglucose
PET: Positron emission tomography
CT: Computed tomography
CRC: Contrast recovery coefficient
BV: Background variability
CNR: Contrast-to-noise ratio
ESD: Emission scan duration
AC: Activity concentration
ALARA: As low as reasonably achievable
ID: Internal diameter
TBR: Target-background activity ratio
BMI: Body mass index
LSO: Lutetium oxyorthosilicate
LYSO: Lutetium-yttrium oxyorthosilicate
PSF: Point spread function
FWHM: Full width at half maximum
NEMA: National electrical manufacturer association
IEC: International electrotechnical commission
b-NEMA-IQ: NEMA-IQ phantom wrapped with the belt
OSEM: Ordered subset expectation maximization
